# Leadership in Orthopaedic Surgery: A Survey of the Value of Leadership Development for Orthopaedic Surgery Faculty

**DOI:** 10.5435/JAAOSGlobal-D-21-00119

**Published:** 2021-10-27

**Authors:** Sean C. Clark, Cadence Miskimin, Mary K. Mulcahey

**Affiliations:** From the Tulane University School of Medicine (Dr. Clark) and the Department of Orthopaedic Surgery, Tulane University School of Medicine (Dr. Miskimin and Dr. Mulcahey).

## Abstract

The purpose of this study was to survey current orthopaedic department chairs at ACGME-accredited orthopaedic surgery programs in the United States to determine whether department chairs valued the importance of formal leadership training and, if so, to understand the leadership development opportunities available along with the benefits of these program for their orthopaedic faculty.

**Methods:**

An anonymous online, voluntary, self-administered questionnaire containing 27 questions was distributed to current orthopaedic department chairs at ACGME-accredited orthopaedic surgery programs in the United States.

**Results:**

Thirty-eight responses were received for a response rate of 27.1%. Twenty-three of 38 (60.5%) department chairs believed leadership training is very important for their orthopaedic faculty. Thirty-six of 38 (94.7%) department chairs did not believe that all their current faculty have strong leadership skills. Twenty-eight of 38 (73.6%) respondents have specific training programs for leadership development at their institution.

**Discussion:**

Most department chairs (92.1%) viewed leadership training for their orthopaedic faculty as either important or very important. Seventy-four percent of orthopaedic surgery department chairs surveyed indicated that they had a leadership development program in place, with 59.1% being developed within the past 10 years. Benefits of these programs included improved listening and communication skills and management of staff.

With medicine constantly evolving, establishing strong management and leaderships capabilities is critical for hospitals, especially academic medical centers.^1^ Leadership is a vital skill for physicians because it is critical for delivering quality care, managing cost, improving medical education, and the success of healthcare institutions.^2,3^ Physicians are promoted to leadership positions based on their clinical acumen, but these skills do not necessarily translate to effective leadership or administrative skills.^4^ Jaffe et al^5^ interviewed 24 surgical faculty from several surgical specialties (general, vascular, plastic, thoracic, and transplant) at a large academic medical center and found that respondents acknowledged a deficit in leadership training during medical and resident education. The skills necessary to be a competent and successful leader require systematic training and include persuasive communication, negotiation, financial decision making, team building, and conflict resolution.^4^

To address the increased needs for developing leadership skills among physicians, academic medical centers and medical schools have invested significant resources to create leadership development programs.^6,7^ For example, Drexel University College of Medicine created the Executive Leadership in Academic Medicine program to provide women in medicine and other healthcare fields with extensive coaching, networking, and mentoring opportunities to place more women in senior leadership positions.^8,9^ Similarly, the University of Michigan created a leadership development program specifically aimed at meeting the leadership goals and motivations of their surgical faculty.^5,10^ Finally, the Pediatric Leaders Advancing Health Equity program at the University of California, San Francisco, integrates leadership training alongside clinical training of pediatric residents to lead advances in health equity for children.^11^

Although more and more programs are being developed, few studies have attempted to understand leadership development opportunities specifically for orthopaedic surgeons. Yayac et al.^12^ found that several leadership development programs for orthopaedic surgeons exist; however, the number of programs is not adequate to meet the demand. The purpose of this study was to survey current orthopaedic department chairs at ACGME-accredited orthopaedic surgery programs in the United States to determine whether department chairs valued the importance of formal leadership training and, if so, to understand the leadership development opportunities available along with the benefits of these program for their orthopaedic faculty.

## Methods

After obtaining approval from our Institutional Review Board (2019 to 1699), an anonymous 27-question online survey using Qualtrics (Seattle, Washington; and Provo, Utah) was distributed through e-mail to 148 department chairs at ACGME-accredited orthopaedic surgery programs in the United States. Follow-up e-mails were sent at 2 and 4 weeks after the initial e-mail to encourage more participation. Of the 148 e-mails sent, 8 were returned as undeliverable, leaving a total of 140 e-mails delivered to department chairs. Some questions only appeared based on a participant's response to a previous question (Appendix A, http://links.lww.com/JG9/A167).

Questions included whether department chairs believed their current faculty have strong leadership skills, how important they viewed leadership training to be, and which faculty members (ie, all faculty versus only those in leadership positions) they recommend receive leadership training. Additional questions asked whether the chair's institution has a leadership development program in place for their faculty and, if so, do they believe it is beneficial.

## Results

### Demographics of Department Chairs and Orthopaedic Surgery Faculty

A total of 38 responses were received for a response rate of 27.1%. Most respondents (16 of 38, 42.1%) have been chairs of their orthopaedic department for 5 to 9 years, whereas 13 of 38 (34.2%) have been chairs for 0 to 4 years and 5 of 38 (13.2%) for 10 to 14 years. Twenty-four of 38 (63.2%) had more than 30 faculty members in their department, whereas 7 of 38 (18.4%) had between 21 and 25. Seventeen of 35 (48.6%) spent 41% to 60% of their time on nonclinical duties, whereas 9 of 35 (25.7%) spent between 61% and 80%, and 6 of 35 (17.1%) spent between 21% and 40%. Twelve of 35 (34.2%) of the chairs' institutions are located in the Northeast, 8 of 35 (22.9%) were located in the Midwest, 10 of 35 (28.6%) were located in the South, and 5 of 35 (14.3%) were located in the West. Most chairs (18 of 35, 51.4%) were aged between 56 and 60 years, whereas 5 of 35 (14.3%) were aged between 66 and 70 years and 4 of 35 (11.4%) between 41 and 45 years. Thirty-three of 35 (94.3%) respondents were men, whereas only 2 of 35 (5.7%) were women. Thirty-two of 35 (91.4%) department chairs were White/Caucasian.

### Importance of Leadership Skills

Twenty-three of 38 (61.0%) orthopaedic surgery department chairs believed leadership training was very important for their orthopaedic faculty, whereas 12 of 38 (31.6%) believed it was important. Only 1 of 38 (2.6%) believed leadership training was very unimportant. Most (36 of 38, 94.7%) of the department chairs did not believe that all their current faculty have strong leadership skills. Chairs recommended that Division Heads/Chiefs (27 of 33, 81.8%), Vice Chairs (27 of 33, 81.8%), Program Directors (29 of 33, 87.9%), and Associate Program Directors (21 of 33, 63.6%) receive leadership training. Sixteen of 33 (48.0%) chairs believed that all faculty should receive leadership training. When asked to rate leadership, conflict management, emotional intelligence/self-management, personnel management/team building, and business/financial skills based on order of importance, most respondents ranked emotional intelligence/self-management as the most important skill, followed by leadership, personnel management/team building, conflict management, and business/financial.

### Characteristics of Institutional Leadership Development Programs

Twenty-eight of 38 (74.0%) respondents have specific training programs for leadership development at their institution, whereas 10 of 38 (26.3%) did not. Examples of these programs included Physician Executive Leadership Program, Dean's Teaching Fellowship, and Faculty Leadership Institute. For respondents with leadership development programs at their institution, 13 of 22 (59.1%) of these programs were developed between 2010 and 2020. Nineteen of 25 (76.0%) chairs were actively involved in the development of their leadership programs, whereas 6 of 25 (24.0%) were not. These leadership development programs were not required for all orthopaedic faculty (25 of 25, 100%). Most (18 of 25, 72.0%) of these institutional leadership development programs are offered in-person, whereas 6 of 25 (24.0%) are both in-person and online, and 1 of 25 (4.0%) is online only. Twenty-one of 25 (84.0%) programs had both lecture/didactic and case discussion/interactive formats, whereas 3 of 25 (12.0%) had only a lecture/didactic format, and 1 of 25 (4.0%) had only a case discussion/interactive format. Twenty-one of 25 (84.0%) programs had external guest speakers, whereas 4 of 25 (16.0%) did not.

Ten of 25 (40.0%) respondents have leadership development programs that meet once a month, 6 of 25 (12.0%) meet twice a month, 3 of 25 (12.0%) meet quarterly, and 3 of 25 (12.0%) meet yearly. The duration of these programs varied as 7 of 25 (28.0%) last between 12 and 15 months, 6 of 25 (24.0%) between 8 and 11 months, 6 of 25 (24.0%) between 0 and 3 months, and 4 of 25 (16.0%) between 4 and 7 months.

### Benefits of Leadership Development Programs

**Figure 1:**
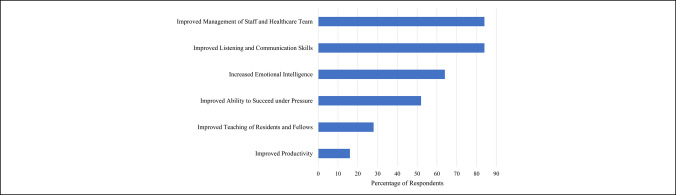
Bar chart showing benefits of leadership development programs.

Eleven of 25 (44.0%) orthopaedic surgery department chairs believed their institutional leadership development program was very beneficial for their faculty, 11 of 25 (44.0%) believed it was beneficial, and 3 of 25 (12.0%) were neutral. When asked to select all the benefits of their leadership development program, chairs chose improved management of staff and healthcare team (21 of 25, 84.0%), improved listening and communication skills (21 of 25, 84.0%), increased emotional intelligence (16 of 25, 64.0%), improved ability to succeed under pressure (13 of 25, 52.0%), improved teaching of residents and students (7 of 25, 28.0%), and improved productivity (4 of 25, 16.0%) (Figure 1).

**Figure 2:**
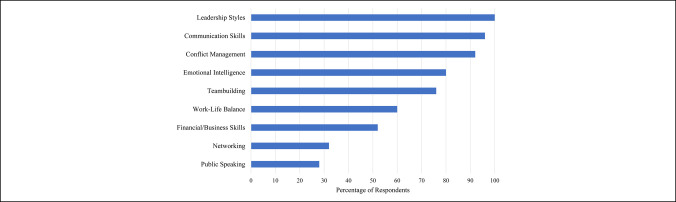
Bar chart showing topics addressed in leadership development programs.

The topics addressed in these institutional leadership development programs included leadership styles (25 of 25, 100%), communication skills (24 of 25, 96%), conflict management (23 of 25, 92.0%), emotional intelligence (20 of 25, 80.0%), team building (19 of 25, 76.0%), work-life balance (15 of 25, 60.0%), financial/business skills (13 of 25, 52.0%), time management (10 of 25, 40.0%), networking (8 of 32, 32.0%), and public speaking (7 of 25, 28.0%) (Figure 2).

### Measuring Effect of Institutional Leadership Development Programs

Department chairs used several methods to measure the effect of their institutional leadership development program including surveying participants (13 of 25, 52.0%), promotion of participants (4 of 25, 16.0%), and evaluating faculty performance (3 of 25, 12.0%). Ten of 25 (40.0%) department chairs reported that the effect of the leadership development program was not measured. All participants of these leadership development programs were allowed to provide feedback regarding the programs.

### External Leadership Development Programs

When asked to select other types of leadership programs that department chairs encourage their faculty to participate in, programs such as American Orthopaedic Association (AOA) Emerging Leaders Program (24 of 35, 68.6%), Association of American Medical Colleges Early Career Women Faculty Leadership Development Seminar (13 of 35, 37.1%), Specialty Society Leadership Training (13 of 35, 37.1%), and AOA North American Traveling Fellowship (11 of 35, 31.4%) were selected (Table 1). Twenty-eight of 35 (80.0%) orthopaedic surgery departments provided funding for their faculty to travel and attend conferences for leadership development. Eight of 28 (29.0%) department chairs provided between $1,501 and $2,000 for traveling and conference expenses, whereas others said they would provide funds as needed.

**Table 1:** External Leadership Development Programs Recommended by Department Chairs for Their Orthopaedic Faculty

Leadership Development Program	Percentage of Respondents
American Orthopaedic Association (AOA) Emerging Leaders Program	68.6
Association of American Medical Colleges (AAMC) Early Career Women Faculty Leadership Development Seminar	37.1
Specialty Society Leadership Training	37.1
AOA North American Traveling Fellowship (NATF)	31.4
AOA APEX Leadership Program	28.6
AOA Kellogg Leadership Series	28.6
Master of Business Administration	28.6
AOA American British Canadian (ABC) Traveling Fellowship	25.7
AAMC Mid-Career Women Faculty Leadership Development Seminar	25.7
AOA-Japanese Orthopaedic Association (JOA) Traveling Fellowship	20.0
Executive Leadership in Academic Medicine (ELAM)	20.0

### Institutions Without Leadership Development Programs

For respondents who did not have a leadership development program at their institution, they were asked if they would be interested in helping to create a program for their orthopaedic faculty. Only 1 of 10 (10.0%) was very interested, whereas 4 of 10 (40.0%) were interested, 4 of 10 (40.0%) were neutral, and 1 of 10 (10.0%) was uninterested. In addition, these respondents were asked why they do not have a leadership development program for their orthopaedic faculty. 2 of 9 (22.0%) believed it was too expensive to develop a program and 2 of 9 (22.2%) it was too time consuming. 2 of 9 (22.2%) department chairs stated that developing a program is not a top priority, whereas 2 of 9 (22.2%) reported using external leadership development programs through the AOA as being sufficient.

## Discussion

In recent years, leadership development programs have become more prominent in academic medical centers throughout the United States. Institutions may be investing more resources in leadership development opportunities for their orthopaedic surgery faculty because 74.0% of department chairs surveyed indicated that they had leadership development programs in place, with 59.1% being developed within the past 10 years. In addition, 80.0% of the department chairs provided funding for their faculty to travel and attend external conferences for leadership development. This is critical because only 5.0% of the respondents believed that all their orthopaedic surgery faculty have strong leadership skills.

A recent study by Pradarelli et al.^10^ evaluated a leadership development program specifically designed for surgeons at the University of Michigan. The authors interviewed 21 surgical faculty who completed the program. Most surgeons reported improvements in their self-empowerment to lead, self-awareness, team-building skills, and business and leadership knowledge. The surgical faculty also reported that the program positively influenced their day-to-day work activities and long-term career development plans.^10^ Similarly, our study demonstrated that 92.1% of orthopaedic surgery department chairs believed leadership development was very important or important for their orthopaedic faculty. Moreover, 88.0% of chairs viewed their institutional leadership development programs as either very beneficial or beneficial. The leadership development program at the University of Michigan along with all of the institutional programs mentioned by department chairs in our study allowed feedback from program participants. With most of these leadership development programs being developed within the past 10 years, allowing participant feedback is critical to promote continued improvement of these programs.

Although most orthopaedic surgery department chairs reported that their institution offered leadership development programs, many still encouraged their faculty to seek and attend external leadership programs such as the AOA Emerging Leaders Program, AOA APEX Leadership Program, and AOA Kellogg Leadership Series (Table 1). A study by Day et al.^13^ studied the effectiveness of the American Academy of Orthopaedic Surgeons Leadership Fellows Program (LFP). The authors compared graduates of this program with applicants who applied to this program but were rejected. They found that graduates of the American Academy of Orthopaedic Surgeons LFP reported significantly higher leadership competency in the areas of knowledge of theory (good knowledge of different leadership styles and aware of own leadership strengths and weaknesses) (*P* = 0.005), tolerance for demands of leadership (comfortable making unpopular decisions and taking responsibility for them) (*P* = 0.001), and leadership positioning (considerate of the ideas of others and integrated into the institution's decision-making process) (*P* = 0.008), whereas competencies such as environmental scanning (good understanding of climate at own institution and can anticipate issues related to education and healthcare delivery) and conflict management trended toward significance (*P* = 0.06 and 0.07, respectively). In addition, graduates of the LFP were markedly more likely to report holding a position of academic rank (instructor, assistant professor, associate professor, or full professor), holding a position of department or subspecialty chief, and/or were chair of a national committee in comparison with applicants who did not complete the program (*P* = 0.02, 0.004, and *P* < 0.001, respectively). Although this study showed the clear objective benefits of an external leadership program, it may be limiting to some physicians because of time constraints, distance, and/or finances.^10^ By contrast, with the onset of the COVID-19 pandemic, external leadership development programs may become more accessible because of the ability to communicate and teach virtually.^14,15^

Of interest, almost three quarters of the orthopaedic surgery department chairs spent between 41% and 80% of their time on nonclinical responsibilities, suggesting that as one advances into leadership positions, more time is spent outside of the clinic and operating room. Similarly, Lobas^16^ surveyed and interviewed 10 internal medicine department chairs at medical schools in the United States and found, on average, 55% of the chairs' time was spent on administrative duties. Another study surveyed 73 chairs of the department of anesthesiology to gain insight into how they manage their department.^17^ The authors found that 39% of chairs' time was spent on administration, committee meetings, and managing people one-on-one, whereas only 31% was spent on clinical care. Thus, as one advances into leadership positions, gaining additional administrative and management skills is important to handle these duties. Institutions should emphasize the importance of leadership and professional development programs for their faculty to become more adept at managing the increased administrative workload because these programs address topics such as conflict management, team building, communication, and financial skills.

This study has several limitations. First, given the relatively low response rate of 27.1%, the results may not reflect the opinions of all orthopaedic surgery department chairs in the United States. Moreover, department chairs who are more enthusiastic about leadership development for their faculty may have been more likely to respond to the survey, leading to response bias. Finally, this is a survey study and, hence, is subjected to the inherent limitations of such a design, including recall bias. Future studies should determine whether leadership development programs should be tailored to individual specialties because different areas of medicine may require a distinct skillset to become a successful leader.
